# Proposed Toxic and Hypoxic Impairment of a Brainstem Locus in Autism

**DOI:** 10.3390/ijerph10126955

**Published:** 2013-12-11

**Authors:** Woody R. McGinnis, Tapan Audhya, Stephen M. Edelson

**Affiliations:** 1 Autism Research Institute, 4182 Adams Avenue, San Diego, CA 92116, USA; E-Mail: sait97302@yahoo.com; 2 Division of Endocrinology, Department of Medicine, New York University Medical School, New York, NY 10016, USA; E-Mail: audhyatk@optonline.net

**Keywords:** autism, nucleus tractus solitarius, blood-brain barrier, autonomic, baroreflex, toxins, hypoxia, perfusion, adrenergic, A_2_ neurons

## Abstract

Electrophysiological findings implicate site-specific impairment of the *nucleus tractus solitarius* (NTS) in autism. This invites hypothetical consideration of a large role for this small brainstem structure as the basis for seemingly disjointed behavioral and somatic features of autism. The NTS is the brain’s point of entry for visceral afference, its relay for vagal reflexes, and its integration center for autonomic control of circulatory, immunological, gastrointestinal, and laryngeal function. The NTS facilitates normal cerebrovascular perfusion, and is the seminal point for an ascending noradrenergic system that modulates many complex behaviors. Microvascular configuration predisposes the NTS to focal hypoxia. A subregion—the “pNTS”—permits exposure to all blood-borne neurotoxins, including those that do not readily transit the blood-brain barrier. Impairment of acetylcholinesterase (mercury and cadmium cations, nitrates/nitrites, organophosphates, monosodium glutamate), competition for hemoglobin (carbon monoxide, nitrates/nitrites), and higher blood viscosity (net systemic oxidative stress) are suggested to potentiate microcirculatory insufficiency of the NTS, and thus autism.

## 1. Introduction

In this article, we present a hypothesis for autism via primary impairment of *nucleus tractus solitarius* (NTS). We propose dual and interrelated mechanisms for impairment of NTS function in the primary pathogenesis of autism. First, the NTS is one of only a few small regions of the brain that fail to fully close openings—“fenestrations”—in the blood-brain barrier (BBB) by one year of age. The BBB of the NTS, or part of the NTS, remains fenestrated permanently, so it allows common neurotoxins that circulate in blood as ions—mercury, cadmium, monosodium glutamate (MSG), fluoride—to preferentially accumulate there. Delayed onset of autism after one year of age could be triggered by exposure to one or more of these toxins, which circulate as ions in the blood and are expected to concentrate in the NTS. Second, there is strong evidence that the human NTS is extremely sensitive to hypoxia/ischemia. Perinatal complications, including newborn encephalopathy, strongly correlate with autism. We propose hypoxic insult to the NTS as a second basis for autism, especially the early-onset variety. In the model we present, exposure to toxins and hypoxia can be independent or interactive causes of autism, because they affect the same brain locus. 

For want of definitive laboratory markers, three unexplained pervasive developmental disorders (“classical” Autism, Asperger Syndrome, and “Pervasive Developmental Disorder Not Otherwise Specified”) are diagnosed solely on the basis of observed behavior. These disorders share core diagnostic criteria: social deficit, impaired communication, and stereotypical or repetitive behavior and interests. Considerable heterogeneity of behavior is observed within each of the three diagnostic groups, and there is substantial behavioral overlap when the groups are compared. In contemporary discussion and research design the three diagnoses often unite in one group as “Autism Spectrum Disorder” (ASD). The terms “autism” and ASD can be used interchangeably, as we do in this hypothesis paper. Abnormal behavior is obvious in the days or months after birth of some children who are later diagnosed as having an ASD. Other children have initially normal progression of social behavior and communication, but then give up the gained behaviors. These “regressions” range from gradual to sudden, and typically occur in the second year. 

Although we have no definitive laboratory biomarkers of ASDs, certain findings point to potential sources of causation. For example, there is a strong trend in epidemiologic studies suggesting an association of ASDs with exposure to atmospheric mercury and cadmium. Highest adjusted odds ratios for ASDs were found in association with estimated atmospheric mercury and cadmium in one study [1], and for estimated atmospheric mercury in a second study [2]. ASDs were also found to strongly associate with atmospheric release of mercury from industrial point sources in a third study [3]. Greater cumulative exposure to mercury in ASDs was suggested by higher total mercury levels in whole baby teeth [4]. Another study found no elevation of mercury in the enamel of baby teeth [5], but enamel is fully formed by 12 months of age while the living dentine of whole teeth is thought to more accurately reflect cumulative mercury exposure until the tooth is lost [6]. 

Higher total mercury has been reported in whole blood [7] and red cells [8] in children with ASD, while a third study found no difference in adjusted levels of total mercury in whole blood [9]. In blood, both organic and ionic mercury have relatively short half-lives; therefore post-diagnosis blood levels may not reflect exposure prior to the onset of ASD years earlier. Greater ongoing exposure to atmospheric mercury might be expected to result in higher ionic mercury in blood of children already diagnosed with ASD, but the blood studies failed to measure the ionic contribution to total mercury. It is important to emphasize that inhalation of atmospheric mercury and cadmium results primarily in the ionic—and thus, BBB-impenetrant—hematogenous states of these metals. Atmospheric mercury is primarily elemental, well-absorbed by inhalation, and is very rapidly converted in blood to the ionic state. Cadmium exists in the ionic state in the atmosphere and remains ionic in blood, and therefore like ionic mercury, is blocked by the BBB due to charge. 

We suggested previously that certain behavioral and biochemical findings in ASDs imply altered function of the brainstem circumventricular organs (CVOs), which fail to develop fully unfenestrated BBB [10]. The CVOs include the area postrema (AP), the pineal gland, the median eminence, and the posterior pituitary. For example, salt-craving, increased water consumption, flavor aversions, carbohydrate-craving, and depressed emesis are reportedly increased in ASDs, and are reproducible in animals via ablation of the AP. Altered sleep patterns and blood levels of melatonin in ASDs imply possible altered function of the pineal gland, which is the sole site of melatonin synthesis. Predominance of an inactive form of circulating oxytocin in ASDs can be considered in the context of axons that traverse the median eminence, or oxytocin storage in the posterior pituitary [10]. If functional problems do exist in the suspected areas of the brain, a common characteristic of these regions is fenestrated BBB. 

From a toxicological perspective, we consider that impaired CVO function may manifest as a *primary* toxicity resulting from the direct effects of exposure to one or more toxins on neurons or glia of the CVOs. The rest of the brain normally develops a mature, unfenestrated BBB by one year of age, but the fenestrations at the CVOs never close. The current assumption is that the BBB develops normally in ASD, as the unfenestrated BBB is not demonstrably leaky in children after diagnosis [11]. The broad class of hydrophilic neurotoxins that circulate in blood as charged ions is thus more likely to have direct contact with the cell membranes of CVOs than other regions of the brain after one year of age. The ubiquity of these hydrophilic neurotoxins in the autism era is indisputable, and it is intriguing to speculate that these primary toxicities affecting the CVOs may contribute to development of ASDs. 

Given the phenotypic similarity of early-onset and regressive ASDs, it is logical to consider how the same toxin or toxins may trigger ASDs by affecting the same region or regions of the brain, but at different times. Lipophilic neurotoxins are not significantly impeded by an unfenestrated BBB, nor by membranes generally, so they could trigger both early and late ASDs via direct toxic effects at the same locus anywhere in the brain. On the other hand, if one or more hydrophilic neurotoxins trigger both early and regressive ASDs after one year of age, the differential effects of the BBB imply that hydrophilic neurotoxins potentially trigger both early and late ASDs via direct effects on CVOs, but not on other regions of the brain. We are aware of no experimental evidence to suggest that exposure to lipophilic neurotoxins results in preferential distribution to CVOs. But, as we will discuss, animal experiments *do* demonstrate preferential accumulation of ionic mercury and cadmium in the CVOs after maturation of the BBB elsewhere. Other hydrophilic neurotoxins may also concentrate in the CVOs, on the same toxicokinetic basis. Thus, evidence suggests that elevated exposures to metals and other neurotoxins in the CVOs do occur, laying the groundwork for the idea that such exposures may contribute to the development of ASDs. 

### 1.1. Chelation as a Clue

Interest in regions of the brain that are unprotected by the BBB increased after initial reports of improvement in behavior of children with ASDs after oral administration of dimercaptosuccinic acid (DMSA) [12,13,14]. As with the hydrophilic neurotoxins, ionic charge renders DMSA strongly hydrophilic [15], and therefore unable to cross the unfenestrated BBB and other lipid membranes. DMSA is well-known to increase urinary excretion of lead (which is not restricted by the BBB), but it also has high binding affinities for mercury and cadmium cations. Therefore, if DMSA in fact acts on the brain to improve behavior in ASDs, it is likely that it does so by affecting one or more regions unprotected by the BBB, such as the CVOs. 

Administration of DMSA to a cohort of children with ASD resulted in significant improvements in behavior, especially verbal communication (*p* < 0.001) and Taste/Smell/Touch (*p* < 0.001) scores [12]. Whether the improvements relate to removal of mercury or cadmium is not clear. Blood and urinary levels of mercury and cadmium, including urinary levels provoked by DMSA, present a mixed picture. After oral zinc supplementation, baseline total mercury excretion exceeded reference range in most subjects with ASD (31/44). A much smaller subgroup (4/44) in the same study had increased baseline urinary cadmium excretion. DMSA challenge of these subgroups significantly increased total urinary mercury excretion (*p* < 0.05) and cadmium (*p* < 0.01) relative to baseline [12]. 

A separate study that did not pre-treat with zinc demonstrated various improvements in ASD behaviors, but different cadmium excretion. Treatment with DMSA significantly increased total mercury excretion of the ASD cohort in relation to reference range, but urinary cadmium did not increase over baseline after DMSA. In fact, while urinary mercury excretion increased relative to baseline, cadmium excretion in urine in the ASD cohort reached a statistically significant *decrease* relative to baseline after multiple treatments with DMSA [16]. Yet another ASD cohort demonstrated significantly *lower* cadmium in whole blood in the ASD cohort versus controls [17]. We submit that these lower cadmium levels do not preclude the possibility of cadmium toxicity in the brain, or in small regions of it.

The different results in urinary excretion of cadmium in the two DMSA studies could be a product of prior supplementation with zinc in the first study, but not the second. Zinc is well-known to induce production of metallothionein (MT), and sufficient unbound MT is needed for mobilization of cadmium [15], which tends to evade removal by DMSA once tissue-bound [18]. Lower levels of unbound MT have not been measured in ASDs, but might be expected on the basis of greater systemic oxidative stress [19] and higher levels of antibody to MT [20]. The binding affinity of ionic mercury for MT is much greater than is cadmium’s for MT. We must consider that lower cadmium in blood and decreasing excretions of cadmium in urine after DMSA treatment could result from higher levels of ionic mercury, in parallel with higher total mercury in blood. 

If it is confirmed, the DMSA effect in ASDs could contribute to either direct or indirect effects on the brain. DMSA administration to children with ASDs rapidly and profoundly increases levels of glutathione [16]. As we will discuss later, glutathione could act systemically as an antioxidant to reduce blood viscosity and enhance microcirculation in one or more regions of the brain. The direct effects of DMSA may include removal of one or more heavy metals from brain cells that are accessible to DMSA, but it also may act by formation of a stable complex that reduces local toxicity of the metal without removing it [21]. If DMSA is acting directly on the brain to reduce the toxic effects of heavy metals, it likely does so at the neuronal membrane level, without entering cells. Heavy metal-induced changes in structure or enzymatic function of membranes could greatly influence neuronal function. For instance, cadmium potently inhibits uptake of the neurotransmitter norepinephrine (NE) in synaptosomes [22]. 

Both *in utero* [23] and in adult rats, cadmium exposure results in abnormal behaviors that associate with increases in non-metallothionein bound cadmium in the liver, kidneys, and small intestine [24]. Chronic exposure of growing rats (with a mature BBB) results in generalized changes in the vascular endothelial bed of the brain and neurodegenerative changes limited to the cerebellar Purkinje cells [25]. Maybe coincidentally, Purkinje changes are considered the most consistent neuropathological finding in ASDs to date. 

Animal experiments show that certain hydrophilic neurotoxins concentrate preferentially at CVOs. In adult rats, intravenous cadmium was shown to accumulate only in regions with fenestrated BBB, as specifically determined in AP and the pineal [26]. Intramuscular injection of adult mice with ionic mercury resulted in accumulation of mercury largely in the AP [27], and a similar experiment in guinea pigs demonstrated that the retention of mercury in the AP was persistent [28]. The findings do not generalize necessarily to all CVOs. However, they are consistent with protracted residence of ionic mercury [29] and cadmium once tissue-bound [30]. An important experiment by Vahter demonstrated that exposure to *organic* mercury, which is not impeded significantly by the BBB, results in very high concentrations of ionic mercury in the pituitary, the one CVO examined. In adult female primates fed organic mercury daily for six months, mercury in the pituitary reached orders-of-magnitude-higher concentration in the pituitary than in six other brain regions and resided primarily as ionic mercury. After organic mercury was discontinued at six months, ionic levels of mercury continued to increase in the pituitary, doubling over another six months. Among these primates, outliers with higher or lower fractions of inorganic mercury were observed, apparently owing to differences in metabolism [29]. 

## 2. Hypothesis

The NTS hypothesis arose in large part from our consideration of toxins and CVOs, and accommodates concomitant toxic impairment of CVOs and the NTS. But NTS impairment more broadly and specifically accounts for the complex set of physical and behavioral abnormalities that associate with ASDs. Physical findings—most conspicuously gastrointestinal and immune—strongly associate with ASDs, and can be assumed to relate to the underlying pathogenesis until proven otherwise. In our experience, parents frequently report synchronous behavioral and somatic changes at time of regression and also during response to therapies. For instance, gastrointestinal symptoms and behavioral regression occur at about the same time [31]. Instead of considering the physical changes that associate with ASD as epiphenomenal, we have chosen to assume that both the physical and behavioral changes reflect primary brain pathology. This “somatobehavioral” model of ASD—in essence both physical and behavioral—is our hypothetical premise, and on that basis we looked for one area of brain that might explain both the physical and behavioral presentation of ASD. 

**Figure 1:**
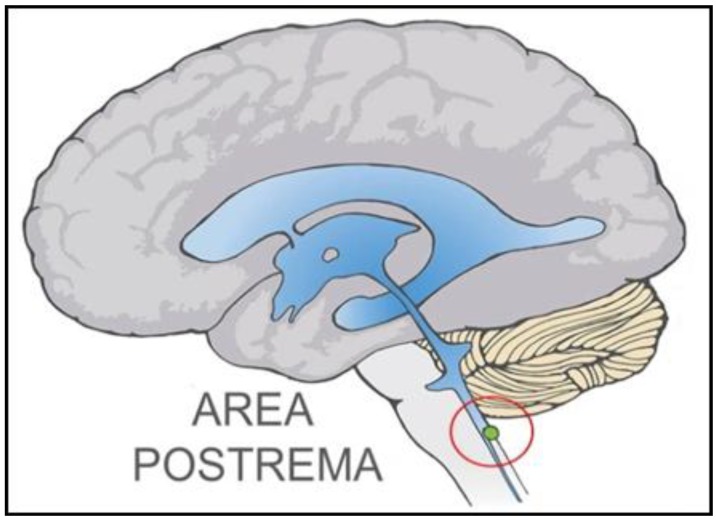
Area Postrema is Anatomical Marker for the Subregion of NTS of Primary Hypothetical Interest. Midline AP is the circled green structure at the lower right. The subregion of the NTS that borders the midline AP is not shown in this sketch, but rather in subsequent figures.

Our hypothesis assigns a primary role in the presentation of ASDs to impairment of the NTS, specifically the subregion of the NTS that cups the ventrolateral aspect of the AP. The location in the brain and approximate scale of the AP, a good anatomical landmark for the adjacent NTS subregion of interest, is represented in Figure 1.

**Figure 2:**
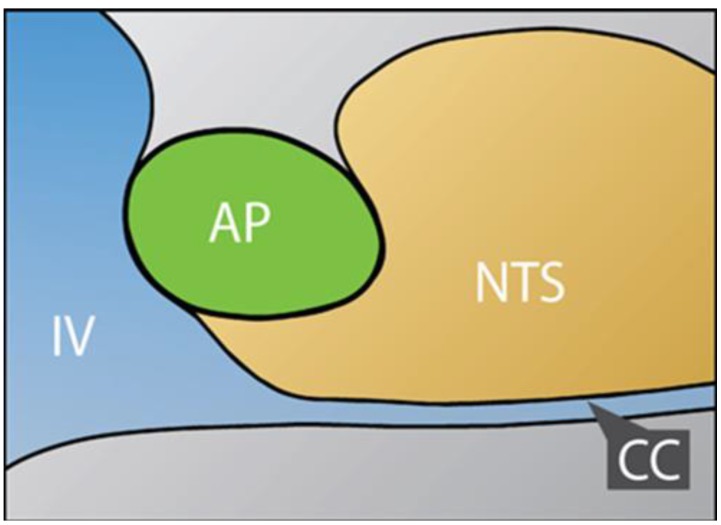
Sketch of a Midline-Sagittal View of Murine AP and NTS. (IV) is fourth ventricle, and (CC) is the central canal, in horizontal orientation. In mice, and presumablyin man, the subregion of the NTS that borders the AP permits neuronal contact with circulating molecules that are excluded by blood-brain barrier in most other regions of brain. The sketch is based on a figure from the Allen Brain Atlas [32].

The NTS is not classified as a CVO, but it is known that the BBB of a subregion of the NTS that is adjacent to the AP remains permanently fenestrated, as in CVOs. Neurons and glia from this subregion of the NTS are expected to have sustained contact with hydrophilic neurotoxins in the blood, and quite possibly progressive accumulation of high levels of such toxins in chronic exposure. The AP and the commissural NTS are true midline structures in humans. Figure 2 is a sketch derived from a microphotograph of murine AP and NTS in the midline sagittal plane. The relationships are expected to be similar in humans. 

Greater uptake in the NTS of neurotoxins that are blocked by unfenestrated BBB is the first of two central elements in the hypothesis. The body of evidence for this vulnerability to uptake of neurotoxins will be reviewed in the next section. The other key element of the hypothesis is selective vulnerability of the NTS to focal ischemia. As we shall see, this vulnerability is strongly implied by unusual gestational anatomy [33] and infarcts confined to the NTS in humans [34,35]. Hypoxic impairment of the NTS, which could interact with the microcirculatory effects of toxins, joins vulnerability to hydrophilic toxins as the second key element of the hypothesis. Inadequate delivery of oxygen to the NTS fits a second strong trend in the epidemiology: that *ASDs are associated with perinatal hypoxia*. 

Fetal distress, maternal hypertension, prolonged labor, cord complications, low Apgar score, and Caesarean delivery associate with ASDs [36] and entail increased risk of fetal hypoxia. In fetal distress, for instance, progressive loss of beat-to-beat variability during labor associates with fetal asphyxia [37] and possibly reflects acute impairment of fetal NTS. Progressive fetal hypoxia is suggested to explain an observed three-fold increase of ASDs in pregnancies complicated by preeclampsia [37,38]. Low fetal pH associates significantly with increased incidence of ASDs, and implies perinatal hypoxia [39]. Newborn encephalopathy has been found to associate with a six-fold increase in ASDs [40]. 

The hypothesis incorporates diverse environmental factors acting at different life stages on the same crucial brain structure. It reconciles the phenotypic similarity of early-onset and regressive ASDs, presentations in subjects outside the diagnostic age range [41,42] and the 20–24-day gestational window for ASDs associated with thalidomide. Aside from brainstem nuclei, very few neurons have formed by the fourth week of gestation [43]. Gestational days 20–24 in humans correspond to days 11.5–12.5 in rats [44], in which electrical activity in response to vagal stimulation begins in the NTS on day 13 [45]. Two major studies have found that maternal folic acid supplementation associates with a significant reduction in the incidence of ASD [46,47], but only if supplementation occurred during the first month after conception [47]. Thus, the time frames for both the thalidomide and folic acid effects correspond to the formation of the NTS. As a prelude to discussion of the microcirculation of the NTS, we will mention here that the basis of thalidomide teratogenesis—including brain—is altered microvascular formation [48] via blocked migration of endothelial cells [49]. 

### 2.1. Dual Vulnerability of the NTS to Toxins and Hypoxia

The concept of dual vulnerability of the NTS to toxins and hypoxia/ischemia is central to our hypothesis addressing the potential etiology of ASDs. A wealth of evidence supports the concept.

**Figure 3:**
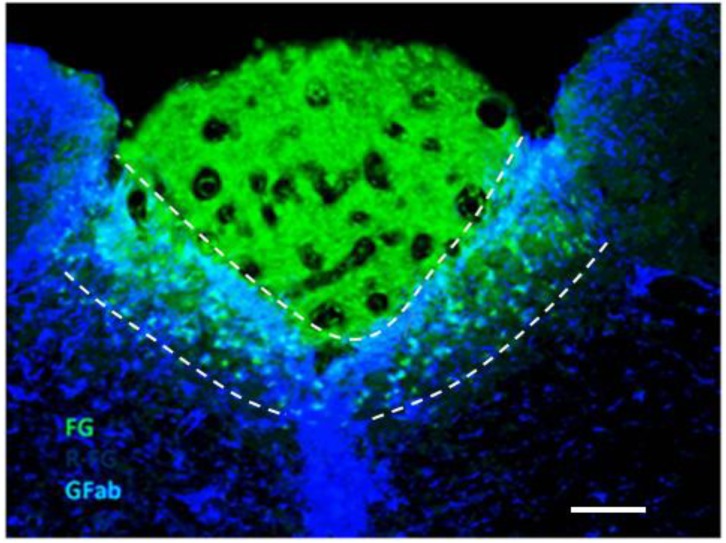
Demonstration of the pNTS with Fluorogold^TM^. The area between the dashed lines in which both green and blue are seen is the pNTS. Fluorogold^TM^ appears as green in this cross-section of rat brain. Blue is immunostain for glial fibrillar acidic protein (GFAP)-positive astrocytes, which are found in the NTS but not the AP. The green triangular area above the pNTS is the AP, which does not stain blue for astrocytes. The area below the pNTS is surrounding NTS that does not exhibit green for Fluorogold^TM^. The scale bar at the lower right is 200 microns. Photo and technical description were provided for original use in this article by Linda Rinaman, Ph.D., Department of Neuroscience, University of Pittsburgh.

Tracer studies demonstrate single-circulation uptake of dye incapable of BBB-transit in the region that corresponds to the *commissural* and *dorsomedial* subnuclei of the NTS [50,51]. Accordingly, robust production of tumor necrosis factor-alpha (TNF) occurred acutely and selectively in the various CVOs and within the commissural and dorsomedial subnuclei of the NTS after administration of bacterial lipopolysaccharide (LPS/endotoxin) [52], which is impeded significantly by unfenestrated BBB. An experiment performed Rinaman localizes this area of rapid uptake. An adult rat was injected with Fluorogold™ (hydroxystilbamine), a fluorescent marker that poorly transits unfenestrated BBB, and was then sacrificed 15 min after injection and immediately brain-sectioned. As shown in Figure 3, a subregion of the NTS that borders the AP demonstrated strong uptake of Fluorogold™, which was not visible elsewhere through the full rostro-caudal extent of the NTS. We suggest a functional designation—“permissive NTS” (pNTS)—for this subregion. The pNTS derives from portions of the commissural and dorsomedial subnuclei of the NTS, comprising 8.2% of total surface ares of NTS at the level of the AP by Rinaman’s calculation.

Inference about the pNTS in humans is based on animal studies, but the 10 subnuclei of the NTS are known to enjoy a high degree of homology in mammals [53]. Electron microscopy demonstrates fenestrations of capillaries in the dorsomedial but *not* commissural subnucleus [50]. Absent immunoreactivity for BBB markers is noted only in the dorsomedial NTS [54]. Rapid uptake also by the commissural NTS has been proposed to result from continuity of Virchow-Robin perivascular spaces with the AP [55]. 

**Figure 4:**
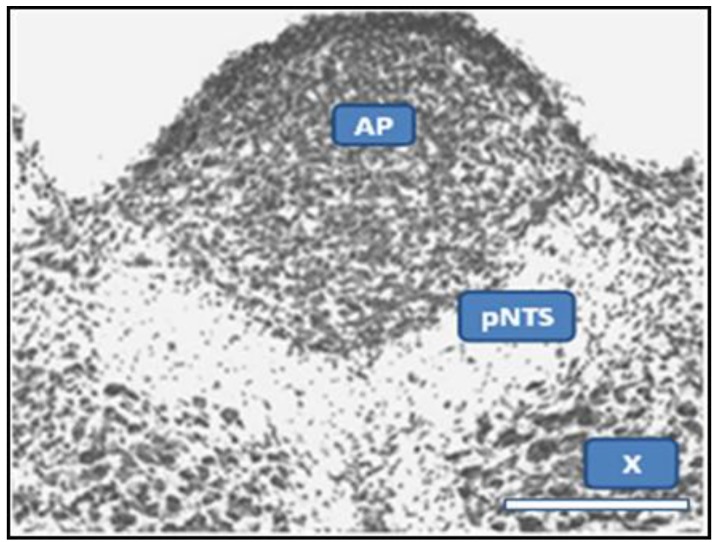
Monosodium Glutamate Effect on the pNTS. This cross section at level of AP from rat given MSG demonstrates preservation of neural elements in the AP and dorsal motor nucleus of the vagus (X), in contrast to total obliteration in the region below AP which corresponds to pNTS. The scale bar at the lower right is 400 microns. Photo from [56], by permission of Literatura Medica, Belgrade.

Commissural axons are in fact shown to extend to the perivascular space of the AP [56], and retrograde transport of impenetrant proteins from fenestrated to unfenestrated brainstem nuclei is described elsewhere (median eminence/arcuate nucleus of hypothalamus) [57]. It is noted that the arcuate nucleus [58] and the pNTS [59,60,61] are preferentially sensitive to MSG, as illustrated in Figure 4.

Vulnerability of the NTS to hypoxia/ischemia is supported by autopsy reports. Adults experience symmetrical brainstem infarcts limited to the NTS in association with hypoxia [35] and hypotension [34]. Both adults and children who died of hypoxia/ischemia had a greater apoptosis index in the NTS than in the adjacent dorsal motor nucleus of the vagus (DMV) and nearby hypoglossal nucleus (XII) [62]. These clinical reports did not consider exposure to toxins. 

Density of N-methyl-D-aspartate (NMDA) receptors correlates well with regional brain sensitivity to ischemia [63]. Forty percent of vagal afferent terminals and 42% of dendrites in the NTS contain NMDA receptors [64], which are found in all subnuclei of NTS.

Ischemic impairment of the NTS without infarction has long been suspected in distressed neonates [65]. In newborns, microvessel density is significantly less in the NTS than in the DMV (*p* < 0.001) [62]. Relative metabolic rates for the two nuclei are not reported for newborns, but they are equivalent at five months [66]. Infarcts of the NTS apparently do not occur in isolation in fetuses and neonates, but segmentally within the dorsomedial brainstem tegmentum served by terminal branches of the basilar artery [65]. Moebius syndrome is found in children who survive such “watershed” infarcts, and Moebius associates strongly with ASDs [65]. 

**Figure 5:**
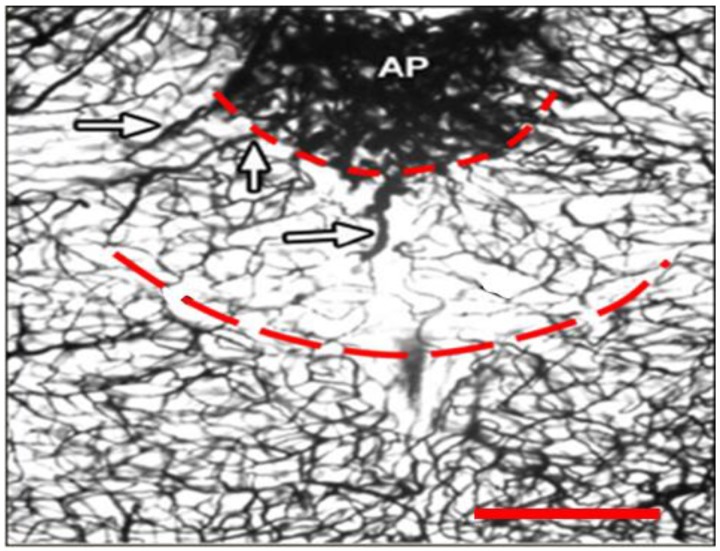
Capillaries of the pNTS in the Rat Brain. Lower capillary density, particularly midline, is visible in the pNTS, the zone between dashed lines, in this cross-sectional view. Arrows indicate short vessels that carry “re-entrant” venous blood to capillaries of the pNTS after prolonged residence in the AP. The scale bar is 400 microns. Photo from [56], by permission of Literatura Medica, Belgrade.

In humans, the commissural NTS is a true midline structure [53,67], configured by rostral-caudal convergence of the right- and left-NTS at the level of the AP [33]. Vascular anastamosis across the sagittal midline of the medulla does not occur during gestation. This effectively limits the commissural NTS to end-vascular supply. Capillary density of the commissural NTS in adult rats was significantly lower than the adjacent medial subnucleus of the NTS and the DMV [55]. Direct visualization of the commissural NTS of adult rats suggests lesser capillary density and minimal anastamosis across the midline, as is evident in Figure 5. Blood supply to the pNTS is described as unusual, with short vessels from the AP that carry “re-entrant” venous blood to the pNTS capillary bed [56]. Prolonged residence of venous blood in the AP [55,68] therefore might favor low oxygen saturation in the pNTS capillary bed.

Thalidomide is not the only neurotoxin that associates with greater incidence of autism and that is also known to alter microvascular formation. Cadmium neurotoxicity in most animal experiments is shown to involve the blood vessels [25] whether the exposure is fetal, neonatal, or after closure of the BBB. Prenatal exposure of rats to cadmium resulted in spheroidal vacuolization of endothelial cells of brain capillaries [69]. Young animals have been shown to be more sensitive to the neurotoxic effects of cadmium [25], and vacuolization of endothelium after exposure of neonatal mice to cadmium primarily affected immature, partially differentiated capillaries [70]. It is not so inconceivable that both cadmium and thalidomide attenuate the development of the microvasculature of the pNTS, and thus lower the threshold for focal hypoxic impairment.

### 2.2. Potentiation of Hypoxia by Toxins

The vasodilatory effects of acetylcholine (ACh) and NE observed in other brain regions are presumably operative in the pNTS. ACh and NE actually have relatively weak direct vasoactive effects, but influence levels of nitric oxide (NO), the principle—and potent—cerebral vasodilator [71]. ACh inhibits, and NE stimulates, release of NO [71]. Acetylcholinesterase (AChE) eliminates ACh, so both AChE and NE are pro-dilatory. The capillaries of the pNTS are distinguished from nearby structures by strong staining for AChE [56], which is suggested to arrive via axonal transport from the AP [72]. 

The A_2_ neuronal subgroup is the source of NE in the pNTS. As catecholaminergic neurons, the A_2_s are tyrosine-hydroxylase (TH)-positive. The A_2_s of the NTS are preferentially sensitive to experimental hypoxia [73]. A_2_ sensitivity to hypoxia and the seminal role of A_2_s in the ascending noradrenergic system are treated in an ensuing section of this paper. 

A number of toxins—including ones that accumulate preferentially at the pNTS—depress AChE function. Ionic mercury [74], cadmium [75], and organophosphate insecticides [74] inhibit AChE, and ionic mercury and cadmium have been shown to disrupt endothelial function [76]. MSG treatment results in excitotoxic degeneration of axon terminals in the pNTS and disappearance of AChE from pNTS capillaries [56]. 

Ingested sodium nitrite reduces brain AChE activity in rats [77]. By converting hemoglobin to methemoglobin, nitrite potentiates hypoxia by depressing the oxygen-carrying capacity of the blood. Nitrites transit the placenta [78] and the breast [79]. Sodium nitrite is a preservative in foods that are often consumed by weaning toddlers, such as baby food, hotdogs, lunch meats, bacon, and ham [77]. Contamination of well water by nitrite/nitrate in fertilizer is not uncommon [80].

Elevated blood levels of nitrite (and nitrate) in ASDs are well-documented [81,82,83] and are usually considered in the context of oxidative stress and inflammation [19]. Irreversible EEG and behavioral changes result from sodium nitrite exposure in animals [80]. Subtle signs of methemoglobinemia—bluish lips, nose and ears—are easy to overlook in early infancy, when intestinal flora that reduce ingested nitrate to nitrite are more likely to trigger methemoglobinemia [84]. A specific antibiotic-resistant bacterium, *Desulfovibrio*, associates with regressive ASD [85], and is known to reduce nitrate [86]. 

The association of ASDs with proximity of maternal residence to freeways [87] stimulates interest in carbon monoxide (CO), which impairs oxygen delivery. Household CO increases with inadequate ventilation of gas appliances, and is observed to sustain for hours at surprisingly high levels (50–100ppm) in living areas of homes after vehicles with catalytic converters are started on cold days in attached garages [88]. Prenatal exposure to another air pollutant, ozone, reduces TH expression in the NTS [89]. 

A broad range of toxins induce oxidative stress [19], which lessens red cell fluidity [90] and thereby increases blood viscosity [91]. In humans, brain capillaries are smaller in diameter than red cells. Deformability of red cells is determined by internal viscosity, including membrane fluidity [92]. Oxidative markers are increased in blood [19], and red-cell membrane fluidity is significantly lower [93] in children with an ASD. Blood viscosity increases vascular shear stress, which in turn activates release of vasoactive mediators from endothelium [94,95] and platelets [96]. While blood viscosity is unstudied in ASDs, urinary markers for endothelial and platelet activation are elevated [97]. The phenomenon of “febrile lucidity” in ASDs [95] could relate to blood viscosity.

We have presented the basis for dual vulnerability of the pNTS to toxins and hypoxia, and mechanisms for toxic effects on oxygenation of the pNTS. Next, we discuss findings that imply that pNTS impairment exists in ASDs and also fits the broad somatobehavioral terrain of ASDs.

### 2.3. Abnormal Baroreflex and Associated Cardiovascular Signs in ASDs

To provide further context for our hypothesis, we will briefly review relevant autonomic mechanisms. The NTS is the central synapse in baroreflex, which, as one of the body’s homeostatic mechanisms for maintaining blood pressure, rapidly adjusts blood pressure in relation to posture and breathing via direct projection to parasympathetic and sympathetic motor nuclei. The NTS receives incoming sensory information from peripheral baroreceptors via the vagus nerve. In response to an increase in blood pressure, lower heart rate (HR) results from increased signals from the NTS to the nucleus ambiguus (NA) and cardioinhibition via the vagus nerve. An efferent arc from the NTS to the sympathetic motor nuclei of the caudoventrolateral and rostroventrolateral medulla reduces tonic vasoconstriction [98]. Mechanical or chemical ablation of the NTS at the level of the AP abolishes the baroreflex, which is impaired experimentally by single doses of oral cadmium [99] or chronic ingestion of ionic mercury [100].

Ming *et al*. studied reduced cardiac parasympathetic activity in children with autism. The investigation applied stringent criteria for adequate relaxation of subjects. At rest, lower cardiac vagal tone (CVT, measured as pulse-interval variability on continuous EKG), lower cardiac sensitivity to baroreflex (CSB, measured as beat-to-beat slowing of HR in response to increase in systolic blood pressure), higher diastolic blood pressure (DBP), and higher mean arterial pressure (MAP) were found in ASDs. The findings were not subtle: 75% of ASD children had a CVT below the control range, 70% had a CSB below. Significance levels for elevated HR and MAP were *p* < 0.001 and *p* < 0.01 [101]. 

Depression of CVT can result from impairment of any component(s) of the baroreflex arc: baroreceptor, afferent nerve, brainstem nuclei, or efferent nerve. CSB is regulated tonically at the level of the NTS [102], so in conjunction with depressed CVT, depressed CSB strongly suggests site-specific impairment of the NTS [101]. Higher resting HR, DBP, and MAP in the majority of ASD subjects is consistent with elevated baseline sympathetic tone [101]. In summary, the Ming study found in the ASD group: (1) depressed baroreflex; (2) decreased vagal (parasympathetic) tone; (3) increased sympathetic tone; and (4) site-specific impairment of the NTS. 

The tonic inhibitory effect of the NTS on the sympathetic nervous system [103] is demonstrated by effects on BP. Chemical stimulation of the NTS lowers BP, and bilateral lesion elevates BP [104]. Clinical findings in ASDs—sleep disturbance, higher skin conductance, larger tonic pupil size, higher respiratory rate [105], and hyperactivity [106]—are consistent with autonomic imbalance. A recent study suggested that heart rate, electrodermal activity, and skin temperature measurements point to an atypical autonomic response to anxiety in ASDs that is consistent with sympathetic over-arousal and parasympathetic under-arousal [107]. Propranolol, an adrenergic antagonist that is used to treat states of hyperarousal, improved speech [108,109] and social behaviors [108] in ASDs. Clearly, the physical parameters and the clinical presentation of ASD are consistent with altered autonomic activity. 

It is important to recognize that NTS is the primary central synapse for viscerosensory input, and that afferent projection to the NTS tends to segregate viscerotopically, with significant overlap at level of the AP [110]. Cardiovascular baroreceptor afferents terminate in the dorsomedial [110,111] and commissural NTS [112] at the level of the AP [113]. This “baroreceptor zone” corresponds substantially to the pNTS. It is not known whether vessels of the brain also send afferents directly to the pNTS. 

Afferent projections of specific viscera to the pNTS are documented in the literature, as seen in Table 1 below. Dense projections for gastric distension are described at the commissural subnucleus [114] and also project to the dorsomedial NTS [115]. Laryngeal sensory terminals are found to cluster most densely at the NTS at the level of the AP [116], and myelinated laryngeal afferents to occur in the commissural NTS [117]. Splenic projections are unreported. Gustatory afference projects to the NTS well rostral to the pNTS, but selective lesion of the commissural NTS results in increased water and salt intake [118], and increased water [119] and salt [10] intake are reported in children with ASDs.

**Table 1:** Afferent Projections to pNTS and ASD Findings.

Viscera	NTS Viscerotopy	ASD Presentation
Baroreceptive	Dorsomedial [110,111] and Commissural [112,113]	Depressed Baroreflex [101]
Gastric Stretch	Dorsomedial [115] and Commissural [114]	Esophageal Reflux [120]
Laryngeal	“Level of AP” [116], and Commissural [117]	Altered Tone [121] and Regression-associated Whisper [10]
Intestinal	Commissural [114]	Retained Paneth Secretions [120]
Splenic	Undetermined	Inflammation [122,123,124]

### 2.4. Suggestion of a Broad Role of the NTS in ASDs

Review of the relevant literature suggests that the NTS plays a broad role in ASDs. As early as 1976, Porges proposed a key role for autonomic dysregulation in ASDs [125,126]. In a series of studies by Porges, cardiac vagal tone measured as “respiratory sinus arrhythmia” (RSA) was lower in ASDs, and associated with social engagement as reflected by gaze [127], social skill ratings, fewer behavioral problems [128], attention [129], emotion recognition [130], and receptive language ability [129]. Changes in social behavior of voles after chronic cadmium or ionic mercury [131] exposure present a model for autistic regression via toxic effects on the brain unprotected by the BBB. 

In 1999, improvements in social interaction, vocalization, and potty training were reported in a series of ASD children who received intravenous secretin [120]. Benefits were too seldom (efficacy in 5/17 trials [132]) or too fleeting [133] for commercial application, but the basic science that paralleled clinical trials strongly associates secretin and the NTS. Secretin binding [134] and mRNA expression [135] in brain sections were found to be higher in the NTS than any other region of the brain, and most NTS neurons were activated by secretin [136], a peptide with limited ability to transit the BBB. 

Behavioral scores reportedly improve after secretin administration only in association with increased biopterin in cerebrospinal fluid (CSF) [137]. Biopterin associates with synthesis of the catecholaminergic neurotransmitters—including NE—produced by TH-positive neurons. Intraventricular secretin activated TH-positive neurons specifically in the dorsomedial and commissural subregions of the NTS [136]. In animals, intravenous secretin increases cerebral blood flow (CBF) [138]. 

Abnormal CBF is one of the better-documented features of ASDs, and could have far-reaching effects on brain development and behavior. Extensively reviewed [139] neuroimaging studies demonstrate bilateral hypoperfusion of temporal [140] and frontal lobes [139], a condition considered to be consistent with global dysregulation of CBF [141]. Asymmetrical temporal and frontal blood flow also is prominent in ASDs [141]. A technical requirement for neuroimaging—motionless recumbency—could hide the full effects of physical activity and posture on CBF. 

The brain is protected from wide deviations in blood pressure by autoregulatory modulation of cerebrovascular resistance [142]. Autoregulation also compensates for changes in blood viscosity [142]. By a mechanism independent of baroreflex, lesion of the commissural NTS is shown to globally impair autoregulation of CBF [103]. 

As determined by ultrasonography, blood flow to the auditory cortex during sound stimulus *decreased* in ASDs [143]. The finding suggests inversion of normal neurovascular coupling, which matches local blood flow to metabolic activity. A parasympathetic influence on CBF [144] and neurovascular coupling [145] is recognized, and is understandable on the basis of NTS projections to the pons. NTS neurons synapse directly with pontine preganglionic parasympathetic neurons, which project to the pterygopalatine ganglia to mediate tonic dilatory effects on the cerebrovasculature [146]. Baroreflex contributes to central vasodilatory tone as effected by this pathway [147], and protects the brain from experimental ischemia [148]. Taken together, these findings suggest that the NTS—or more specifically, the pNTS—is important in the regulation of CBF. 

Vagal nerve stimulation (VNS) enhances neurovascular coupling [149]. VNS as a potential treatment for ASDs was suggested by experiments with a cerebellar-lesioned animal model that demonstrated NTS modulation of exploratory behavior [150]. Focal electrical [151] or chemical [152] stimulation of the commissural NTS increases CBF and synchronizes EEG. Seizure suppression by VNS [151] depends on activation of vagal afferents to the NTS [153]. Along with seizure suppression, VNS appears to improve behavior in some children with ASDs. “Striking improvements” were reported in four subjects with severe autistic behaviors after VNS for seizure control [154]. Another study examined behavior of eight children with ASDs and seizures two years after initiation of VNS; just three had improvement in general function, and none had positive cognitive effects [155]. Examination of a larger ASD cohort twelve months after VNS placement suggested improved neurocognitive performance, particularly alertness [156]. 

VNS underscores basic vagal anatomy: the vast majority of vagal fibers are afferent, and the vagal afferents primarily synapse in the NTS. A block or attenuation of the flow of sensory information from viscera to brain—“deafferentation”—could result from lesion of incoming vagal fibers, or from lesion of the NTS relay point. As will now be presented, experimental deafferentation of viscera results in significant and specific changes in visceral function, and it is plausible that the specific changes in visceral function seen in ASDs result from deafferentation. 

## 3. Visceral Deafferentation Matches the ASD Phenotype

The profile of baroreflex and cardiovascular signs in ASDs from the Ming *et al*. study is essentially mirrored by electrolytic lesion of the NTS at the level of the AP: decreased HR variability, increased BP volatility, and increased HR [157] As would be predicted, transection of the left vagus (vagus is the only asymmetrical nerve in the body) eliminated 90% of the baroreflex [158]. The parasympathetic component of the baroreflex is blocked by microinjection of a selective NMDA receptor antagonist [159] into the commissural NTS. Also, selective destruction of A_2_ neurons of the NTS—to be discussed later—has a hypertensive effect [160]. Thus, experimental visceral deafferentation, including focal lesion of the NTS, results in cardiovascular changes also documented in ASDs. 

### 3.1. The Immune System and Deafferentation

It is well-recognized that immunity in ASDs tilts strongly towards production of inflammatory cytokines [122,123,124]. Intriguingly, the response of monocytes to stimulation with phytohemagglutinin is found to associate with aberrant behavior and impaired core behavior of children with ASDs [124]. 

The NTS has been called the “central switchboard for neuroimmune communication”. Autonomic regulation of inflammation involves both synaptic and neurohumeral transmission of information about peripheral immune status to the NTS [161]. The pNTS is accessible to large molecules (cytokines, antibodies) produced in response to immune challenge, and is in fact a primary detector of circulating cytokines for the central nervous system [162]. 

LPS, a potent activator of microglia [163], rapidly enters regions of the brain lacking BBB. Lidocaine injection of the NTS blocks the systemic inflammatory response to LPS, including a surge in plasma TNF [164]. In turn, VNS blocks increase in plasma TNF after LPS [149,164]. Social withdrawal after LPS administration is suppressed and restored in relation to temporary inactivation of the “dorsal vagal complex” [165], which is comprised of the NTS, the AP, and the dorsal motor nucleus of the vagus (X), as shown in Figure 3.

Vagal stimulation restrains production of inflammatory cytokines [166] in response to ischemia, inflammation, and infection [167]. Efferent vagal activity releases peripheral ACh to bind with ACh receptors on monocytes and macrophages to inhibit cytokine release [167]. Mean plasma levels of ACh are reduced by nearly 70% in ASDs [10].

Vagal efferents synapse in the celiac-mesenteric ganglion to release ACh, which stimulates the splenic nerve to release NE in the spleen, which down-regulates inflammatory cytokine production [167,168] via adrenoreceptors on CD3+, CD4+, and CD25+ cells [169]. Splenectomy and selective abdominal vagotomy block the anti-inflammatory effects of VNS [170], increasing the proportion of circulating pro-inflammatory lymphocytes [171]. 

VNS experiments with ACh-receptor knock-out mice demonstrate the role of the splenic afferent signal in the down-regulation of inflammation. After administration of LPS to ACh knock-out animals, VNS depressed TNF. But after transection of the vagus, electrical stimulation of the distal segment did not depress TNF [169]. Within our hypothetical framework, immune dysregulation in ASDs is a consequence of splenic deafferentation by impairment of the NTS.

### 3.2. Laryngeal Function and Deafferentation

The laryngeal muscles are visceral, and vocalization requires autonomic modulation of the larynx [172]. Afferent signals determine movement and strength of the laryngeal muscles [173], and facilitate coordination of species-specific vocalization via the NTS [174]. Thus, selective lesion of the purely-sensory internal branch of the superior laryngeal nerve (SLN) severely impairs vocalization in primates [174]. In pig experiments by Sasaki and colleagues, unilateral section of the SLN, which contains few motor fibers, results in reduction of glottal closing force (GCF) by fully 46% [175].

Acoustically-strong voice is replaced by whisper if GCF is diminished sufficiently [173]. Reduced glottal closing is evident on videolaryngoscopy of patients with Parkinson’s disease (PD) [176] and underlies their hallmark reduction in vocal intensity and speech inaudibility. Whispering PD subjects feel as though they are shouting or exerting great physical effort when asked to speak more loudly [173]. Voice onset errors (unvoiced “P”/voiced “B” substitution) in PD also are explicable on the basis of altered laryngeal afference [173]. Abnormal tonal quality is consistent with vagal alteration [177], and common in PD [173] and ASDs [121]. Earliest evidence of brain pathology in PD is observed in the DMV [178], immediately adjacent to the NTS. 

We were alerted to whisper as component of vocal regression by a child who, but for age of onset, met the criteria for the diagnosis of regressive ASD. 

CASE STUDY: Prior medical history, including pregnancy, delivery, and developmental milestones, is unremarkable for male child RK. The child was seen in private practice by co-author WRM, and the parents requested this publication and confirmed its accuracy. As a pre-schooler, he was described by his parents as inquisitive, talkative, bilingual, and outgoing: “Full of life...like his sister, going to be an honor student”. At age four, under local anesthesia, RK received six amalgam dental fillings at once, including two amalgam root canals. He was described as more sleepy than usual the day of and day after the procedure.

Some days—not weeks—after the amalgams, his parents observed changes in his behavior. Gradually, RK became “withdrawn, less playful” and “less talkative” including fewer and less vivified interactions with family members. Within weeks the parents were gravely concerned by the drop-off in spontaneous and prompted speech. Residual speaking became softer and less audible, and after six-eight weeks, vocalizations ceased. He continued to respond non-verbally to parental communication.

The mother recounts precisely the child’s last spoken words. He whispered to her softly, “I am shouting”, and said nothing more. There is no history of concurrent illness, injury, or medicines in this time frame and the rest of the family remained well. After several weeks without vocalization but considerable parental encouragement, RK began to regain a few spoken words. In addition to vocal paucity, a specialist evaluation at five years also described poor social reciprocity, sleep disturbance, and touch- and sound-sensitivity. Diagnosis of Landau-Kleffner syndrome was proposed in absence of characteristic EEG changes.

At six years, secretin treatment associated with increased speech. By six years and eight months, the child’s effective vocabulary, still modest in comparison to his pre-regression status, had increased to about one hundred words. Gilliam Autism Rating Scale at this time was consistent with ASD. Mercury elevations in RK’s serum, hair, and urine were attributed to amalgams by a consulting pediatric neurotoxicologist. Lymphocytic testing by the Karolinska Institute in Stockholm demonstrated increased sensitivity to mercury.

At seven years all six amalgams were extracted at once, under general anesthesia, without a protective well, high-suction, or pharmacological prophylaxis. The child spoke once on the evening after the procedure, “Band-aid”, made a few sounds the next day, and then ceased all vocalization for weeks afterwards. At seven years and nine months, the child was described by his parents as more alert, focused, and “happy” after treatment with oral DMSA.

At fifteen years, pubertal RK was compliant with simple instructions by his parents, highly limited in interaction with strangers, and moderately gaze-averse. His vocalizations were limited to single words or very short phrases, and he reportedly struggled to read aloud first-grade books. Parental optimism about eventual self-sufficiency remained modest. Introduction of a keypad communicator resulted in responses more sophisticated than expected, such as identification of the president by name.

In the child RK, loss of vocalization and social function occurred gradually in temporal relationship to initial placement of mercury amalgams, and then again dramatically and immediately after extraction, which is known to acutely increase blood-borne mercury [179]. Anesthesia is excluded as a co-variable. By description, the regressions are quite similar, and both relate temporally to major amalgam work. It is reasonable to consider that the described regressions were triggered by mercury from the amalgams. Accumulation of ionic mercury from amalgams in areas of the brain that lack full BBB protection is kinetically predictable. Mercury in amalgams reaches the bloodstream as mercury vapor, in the uncharged elemental state. Conversion of elemental mercury to ionic mercury in blood is rapid, and inorganic mercury in blood associates strongly with the presence of amalgams and their removal [179,180]. The rapidity and extent of the second regression can be seen as a consequence of the conversion of a very large pulse of blood-borne elemental mercury vapor to ionic mercury that was subsequently deposited in the pNTS. Lower blood levels of mercury vapor and less rapid deposition of ionic mercury may account for the gradual onset of the first regression.

As discussed earlier, whisper is a hallmark feature in PD, and whisper in PD associates with reduced GCF. RK’s first vocal regression clearly associated with whisper, and the accompanying first-person description appears to us distinctly Parkinsonian, *i.e.*, *seeming as if to shout*. Since whisper in PD associates with reduced GCF, and since experimental deafferentation of the larynx of pigs results in decreased GCF, it is logical to suggest that RK’s whisper had to do with laryngeal deafferentation as a result of impairment of the NTS. 

It was surprising to find that whisper in association with vocal regression is reported in many children with ASDs. The Autism Research Institute parental questionnaire asked about whisper over several decades. Of 23,685 ASD children who had begun talking normally, 17% had “normal talk replaced by whisper” for at least one week. Of those who whispered for at least one week, 42% deteriorated to complete loss of vocalization, and 16% of children continued to whisper long-term [10]. The association of whisper with vocal regression in many children with ASDs increases our suspicion that laryngeal deafferentation via NTS impairment may explain vocal deficits in many cases. 

Assistive communication technology has been reported to unmask higher cognitive functions in some, but not all, children with ASDs. Higher levels of expressive communication are achieved in low-verbal ASDs with keyboards [181] and speech-generating devices [182] that bypass the vocal apparatus. As mentioned earlier, improved speech is reported in a majority of ASD children with fever [95], and mild hyperthermia was shown to fully restore GCF in the aforementioned Sasaki pigs after section of the SLN [183]. We suggest that in addition to improvement in effective blood flow in the NTS via lower blood viscosity, increased body temperature may serve to up-regulate a temperature-sensitive receptor found in the NTS. Both NMDA and non-NMDA glutamatergic afferent transmission support laryngeal adduction [184]. Temperature-sensitive vanilloid receptors (TRPV1+) are distributed in afferent terminals of the NTS at the level of the AP [185], and act presynaptically to increase glutamate signaling via potassium channels [186]. A sharp rise in glutamate release and activity of NTS neurons between 37 and 40 °C attributes to upregulation by TRPV1+ [187]. Glutamate release via TRPV1+ is not blocked by cadmium, and is therefore independent of voltage-gated calcium channels [187].

### 3.3. Gastrointestinal Function and Deafferentation

Deafferentation of the gastrointestinal viscera is also suggested in ASDs. Experimentally, vagal deafferentation by microsurgical removal of the nodose ganglion, with preservation of vagal efferents, decreases end-organ secretory function [188]. Retained Paneth cell secretions in the intestine of children with ASDs [120] can be viewed in this light. By the same token, gradual recovery of potty-training skills after regression [189] and rapid restoration of potty-training in response to intravenous secretin [190] might be explained by gradual improvement in viscerosensory transmission. 

Reflux esophagitis in two-thirds of children with ASDs [120] is explicable on the basis of gastric stasis and distention due to deafferentation. As presented earlier, projections for gastric distension are found in the commissural [114] and the dorsomedial [115] NTS, and so are present in the pNTS. Small doses of systemic LPS rapidly delay gastric emptying [191], and microinjection of the NTS with an opioid inhibits gastric motility (and intestinal secretion) [192]. Constipation is common in ASDs [193], and is consistent with depressed peristalsis. More than half of a random cohort of children with ASDs had radiographic evidence of fecal retention, including megacolon [193], as did 100% of a regressed cohort, with or without history of intercurrent diarrhea [194]. 

In our collective clinical experience, a gluten-free diet often relieves chronic constipation in subjects with ASDs. Opioid peptide gliadorphin-7 fails to transit the BBB [195], but opioid receptors exist in the dorsomedial subnucleus of the NTS [196], where BBB remains fenestrated. Microinjection of the NTS with opioid reduces visceral afferent activation of TH-positive neurons by 80% [197]. Opioids inhibit NTS calcium channels [198], and calcium channels also are notoriously sensitive to heavy-metal inhibition. Results of NTS experiments in animals are compared to visceral findings in ASDs in Table 2. 

**Table 2:** NTS Animal Experiments and ASD Findings.

Experiment	Observation	ASD Finding
Electrical ablation	Baroreflex depression [157]	Depressed baroreflex [101]
Chemical blockade	Baroreflex depression [159]	Depressed baroreflex [101]
Selective A_2_ lesion	Blood pressure increase [160]	Higher blood pressure [101]
Commissural lesion	Increased water and salt intake [118]	Higher water [119] and salt [10] intake
Commissural stimulation	Increased cerebral blood flow [151,152]	Cerebral hypoperfusion [139,140,141]
Commissural lesion	Decreased autoregulation [103]	Cerebral hypoperfusion [139,140,141]
Commissural opioid microinjection	Blocks gastric motility and intestinal secretion [192]	Esophageal reflux and unreleased Paneth secretions [120]

## 4. Cognitive Dysfunction Due to Pathology in the A_2_ Neurons of NTS

Function of higher brain centers in ASDs could be subject to disordered regulation of CBF due to impaired autonomic function of pNTS. In addition, higher cognitive function in ASD may involve changes in function of the ascending noradrenergic system, as affected by the contribution of a specific NE-producing neuronal subgroup found in NTS, the A_2_. Noradrenergic neurons of the NTS and the adjacent DMV comprise the A_2_ component of the ascending noradrenergic system. There is experimental evidence to suggest that the A_2_ neurons are more sensitive to hypoxia and are therefore of particular hypothetical interest.

The A_2_s are in essence a seminal point for an ascending brainstem noradrenergic system that begins in the NTS, in the caudal-most brainstem. The noradrenergic system enjoys extensive reciprocal connections with higher brain centers, and A_2_s play a significant role in modulation of affect, learning, memory, and sickness behavior [199]. The A_2_ neurons as a class produce NE. A_2_ neurons lack the enzyme for synthesis of adrenalin, which is produced by their C_2_ counterparts [200].

**Figure 6:**
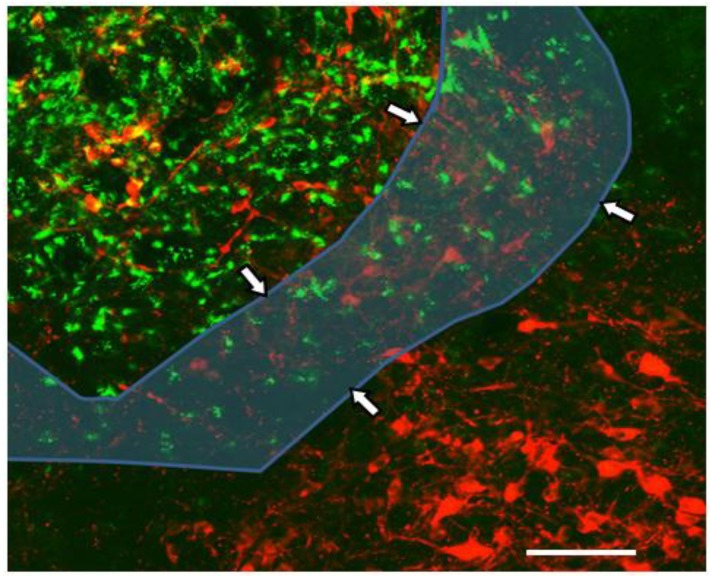
A_2_ Neurons are Found in the pNTS. An off-center cross-sectional close-up of rat brain shows red for immunostain for dopamine beta hydroxylase (DbH), a marker for A_2_ neurons. Fluorogold™ administered intravenously 15 min prior to sacrifice appears green. The pNTS is the area of blue transparency with arrows at the margins. The AP at upper left and the pNTS exhibit both NE-producing cells and Fluorogold™, but Fluorogold™ does not appear elsewhere in the NTS, lower right. The scale bar is 200 microns. Photo and technical description were provided for original use in this article by Linda Rinaman, Department of Neurosciences, University of Pittsburgh.

A precursor enzyme, DbH, is used to identify the A_2_ subgroup of NTS neurons. Microscopic examination of rat brain demonstrates that A_2_ neurons populate the pNTS (See Figure 6). 

A_2_ neurons participate widely in visceral reflex arcs but also relay and integrate viscerosensory information for the ascending brainstem noradrenergic system [199], known in the past as the “reticular activating system” [201]. Rimland proposed altered function of reticular formation in autism as early as 1964 [202]. A_2_s of the commissural NTS are implicated in the normal sickness response [203], which includes anorexia, hyperalgesia, malaise, fever, and adrenocortical response to infection. Experimentally, sickness behavior is induced by intravenous administration of inflammatory cytokine or LPS [204]. In our experience, parents commonly report that their ASD child “just never seems to get sick when the other children do”. Altered sickness behavior in ASDs tends to implicate A_2_s, because sickness behavior is in the A_2_ functional domain.

*A_2_ neurons are preferentially sensitive to experimental hypoxia*. Experimental hypoxia/ischemia in rat pups at postpartum day three resulted in no changes in C_2_ adrenergic neurons of the NTS at 21 days, but A_2_ neuronal numbers declined significantly [73]. In addition, neonatal asphyxia changes catecholamine turnover [205] and response to stress of neurons of the NTS later in adulthood [206]. We can infer from effects in animals that A_2_ catecholamine production might be affected by neonatal asphyxia in humans, and that neonatal hypoxia might also result in selective and persistent changes in human A_2_.

In response to focal hypoxia, the brain normally increases levels of NE [207]. As discussed earlier, NE increases local NO, which is pro-dilatory. Also, release of NE in response to hypoxia/ischemia has been shown to blunt excess glutamate release, thereby reducing the excitotoxic component of hypoxia [207]. This mechanism is well-demonstrated in the pNTS [207]. Glutamate-stimulated NE release by A_2_ neurons of adult animals is depressed after early post-natal MSG exposure, at doses that did not result in morphological changes [208]. Finally, NE—but not dopamine, serotonin, or histamine—increases intracellular cAMP via the alpha-2 receptor, thus increasing extracellular accumulation of adenosine, which dilates blood vessels [209]. If hypoxic—or neurotoxic—impairment of NE production by A_2_ neurons results from perinatal complications, then lesser production of NE is expected to potentiate excitotoxicity and lower the vasodilatory stimulus in the pNTS.

The clinical response to clonidine, an alpha-2 agonist, suggests a central NE deficit in ASDs [210]. Clonidine improved irritability and hyperactivity [211], reduced hyperarousal, improved social behavior [212], reduced impulsivity, improved attention, lessened hyperreactivity, and improved sleep [213] in ASDs. A recent open-label trial of reboxetine, a selective NE reuptake inhibitor, improved depression and ADHD symptoms in adolescents with ASDs [214]. Larger tonic pupil-size in ASD inversely correlates with a marker for peripheral NE [215]. Clonidine is known to act at the NTS to increase cardiac vagal tone [216], so would appear to address a known visceral malfunction in ASD. Behavioral benefits from these drugs presumably relate to enhanced NE levels, which hypothetically could compensate lower NE production by the A2 due to birth hypoxia. 

## 5. An Animal Model for Focal Inflammation of the NTS

Behavioral, physiological, and laboratory findings in ASDs are comparable to those seen in the spontaneously hypertensive rat (SHR). Hyperactive behavior is common in ASDs, and SHRs are utilized extensively as a behavioral model of attention-deficit hyperactivity disorder (ADHD). Sympathetic activity is greater in SHRs [217], and baroreflex is abnormal [218]. Hypertension is not manifest in younger SHRs [219], nor has ambient atmospheric exposure been considered etiologically. Cadmium exposure increases blood pressure and HR of SHRs, and results in greater activation of endothelial phosphokinase C [220]. 

Focal pathology is found in the NTS of SHRs, with marked inflammation and accumulation of white cells within the microvasculature [221]. Leukocytic adhesion is evident [219,221], and associates with greater endothelial expression of the pro-inflammatory junctional-adhesion molecule (JAM-1), which binds white cells and activates platelets [222]. Adenoviral expression of JAM-1 in the NTS of normotensive rats results in increased blood pressure [221]. Inflammation of the microvasculature of the NTS is suggested to impair circulation by increasing local vascular resistance [222]. Expression of the endogenous inhibitor for inflammatory leukotriene B4 (LTB4) is decreased in the NTS of SHRs, and is suggested to contribute to local inflammation [219]. 

Increased peripheral leukocyte adhesion and platelet aggregability in SHRs [221] compare to elevated peripheral markers for endothelial activation (6-keto-prostaglandin F[1alpha]) and platelet activation (2,3-dinor-thromboxan B[2]) in ASDs [97]. Calcitonin gene-related peptide (CGRP), thought to carry sensory signals to A_2_ neurons of the NTS [56], is an unusually potent vasodilator [223]. CGRP is elevated vastly both in the blood of SHRs [224] and of neonates who are eventually diagnosed with an ASD [225]. Chemokine (C-C motif) ligand 5 (RANTES) expression is down-regulated in the NTS of SHRs [219] and also is depressed in unadjusted estimates from blood spots of neonates later diagnosed with an ASD [226].

Focal inflammation is a predictable consequence of local ischemia and/or deposition of inflammatory toxins at the pNTS. Ischemia/hypoxia is known to trigger microvascular inflammation [222,227]. Late gestational intrauterine asphyxia of minipigs results in microglial activation limited to the brainstem [228]. Microglial activation is associated with many toxic exposures [229]. Increases in microglia occur after exposure to ionic mercury [230] and in association with ionic mercury accumulation in CVOs after ingestion of organic mercury [29]. Low concentrations of cadmium potently stimulate inflammatory cytokines [231]. MSG increases TNF in brain unprotected by a BBB [232]. LPS initially induces TNF only in CVOs, followed by a migratory pattern of TNF-positive microglia around the CVOs at three hours, then widespread TNF-positive cells at six hours [233]. 

In ASDs, TNF is elevated in CSF [234] and central in origin [235]. A specific microanatomic feature suggests a mechanism whereby focal inflammation of the NTS could contribute substantially to elevated TNF in CSF. Besides lacking a BBB, the AP lacks tight junctions with CSF [57], and as referenced earlier, the AP and the NTS share bi-directional axonal projections. The microanatomy therefore would appear to favor exchange of BBB-impenetrant molcules between pNTS and CSF. 

Neurons of the NTS are highly responsive to inflammatory cytokines [204]. An increase in local cytokine levels is shown to alter function of the NTS. Specifically, TNF alters glutamate release by primary vagal afferents of the NTS to produce extreme gastric stasis [236]. ACh is noted to block TNF-induced activation of endothelial cells and recruitment of white cells [237]. 

Pericytes are the contractile unit in brain capillaries, which do not contain smooth muscle. Pericytes regulate blood flow [238], probably in relation to neural activity, by constriction or by stiffening processes that affect the passage of deformable red cells [239]. Platelet-derived growth factor (PDGF) knock-out mice fail to develop brain pericytes, and have been offered as a behavioral model for ASDs [240]. PDGF blood levels in ASDs are depressed, and relate to clinical severity [241]. 

Contractility of pericytes is affected by ACh [242] and NE [239], which have opposing effects on local NO release, as discussed earlier. NO has been shown to relax pericytes [243], but excess NO is toxic to them [244]. The NTS is distinguished by the highest density of adenosine receptors in the brain [245], and adenosine is known to exert vasoactive effects on pericytes [246]. Adenosine normally accumulates in response to ischemia [247], which is certainly compatible with earlier discussion of adenosine as vasodilatory [209]. As we hypothesize, hypoxic—or toxic—impairment of A_2_ could result in lower NE production and therefore lesser local adenosine production. Lower adenosine is expected to hamper normal vasodilatation in the pNTS, and the proposed effect is via pericytes. The temperature-sensitive receptor TRPV1 that was discussed earlier as a possible mechanism for febrile lucidity is found in pericytes [248]. 

Altered potassium currents may play a role in impaired function of the pNTS in ASDs. Adenosine is known to modulate potassium currents in the retina [246], and therefore possibly in the NTS as well. In any case, TRPV1 as found in pericytes and is known to activate potassium channels in the NTS specifically [186]. It is also known that experimental inversion of neurovascular coupling is mediated by activation of potassium channels [249]. These data suggest that possible inversion of the neurovascular couple in ASDs [143] could result from altered potassium currents in the pNTS, as mediated by adenosine and pericytes. 

The SHR provides a model for focal pathology of the NTS, including inflammation of the microvasculature of the NTS. We discussed how hydrophilic neurotoxins—mercury, cadmium, MSG—and hypoxia/ischemia elicit a strong inflammatory reaction by microglia, and therefore could be expected to elicit local inflammation in the pNTS as they accumulate there. If A_2_ neurons are part of the suspected pathology of the pNTS in ASDs, then decreased adenosine and consequent pericyte dysregulation of the microvasculature could further potentiate focal ischemia. Altered potassium currents are another possible consequence of abnormal adenosine production and pericyte function in pNTS. 

## 6. Brain Hypoxia in ASDs

As we have proposed, somatobehavioral features of ASDs largely could result from NTS impairment after perinatal hypoxic insult or from later exposure to toxins that hinder oxygenation of the NTS. It follows that sub-clinical perinatal hypoxic effects on the NTS might lower the threshold for toxin-triggered ASDs. Vasculogenic gestational influences (e.g., thalidomide) on microvasculature of the NTS could lower the threshold for either perinatal or toxic triggers, or both. 

Widespread abnormality of the brain outside the NTS is viewed as the neurodevelopmental consequence of perinatal hypoxic insult and/or altered brain perfusion secondary to NTS impairment. Abnormal development of brain regions known to be sensitive to global ischemia is prominent in ASDs. Decreased cerebellar Purkinje cells are perhaps the most consistent neuropathologic finding in ASDs. Purkinje are preferentially sensitive to both global brain ischemia [250,251], and to lactational ionic mercury [252]. Reduced corpus callosum is reported in ASDs [253,254] and experimental neonatal asphyxia [255]. 

Abnormal cortical laminar organization in ASDs [256] is comparable to changes in experimental fetal asphyxia [257]. Reduced neuronal size and fewer superior olivary nucleus (SON) are found in ASDs [258]. SON is preferentially sensitive to experimental birth asphyxia [259,260]. Abnormal brainstem auditory response in infants associates with perinatal asphyxia [261] and incidence/severity of ASDs [262]. Neonatal asphyxia exerts lifelong effects on neuronal responsiveness in the catecholaminergic projecting area of the brain, including the NTS [206]. 

In addition to autonomic effects on cerebral perfusion, NTS impairment could result in functional changes in other visceral systems with downstream effects on oxygenation of the brain. Serotonin (5-HT) elevation in the blood is one of the better-documented and consistent findings in ASDs [263] and is probably gastrointestinal in origin [264]. Paracine 5-HT stimulates intestinal peristalsis by upregulation of vagal afference [265]; therefore greater intestinal 5-HT in ASDs is a possible compensation for afferent block at the NTS. 

Aside from the peristaltic effect, increased intestinal production of 5-HT conceivably supports other lagging NTS functions by increasing circulating 5-HT. While 5-HT does not transit the BBB, 5-HT receptors are found in the pNTS [266]. Serotonin is shown to act in the NTS to facilitate baroreflex [267], to activate catecholaminergic neurons [197], and to increase EEG amplitude [268]. On the potential physiological downside, 5-HT is a relatively potent vasoconstrictor, so elevations in the blood may depress brain perfusion by effects on cerebrovascular endothelium. 

Diffuse effects of chronic hypoxia are suggested by the dendritic signature of ASDs. It is well-known that dendrites are preferentially sensitive to hypoxia, as demonstrated specifically in NTS lesions [34]. An excitotoxic component of hypoxic injury is well-described, and prominently dendritic. NMDA receptor density predicts sensitivity of dendrites to hypoxia, as seen experimentally in the CA1 area of the hippocampus [63], where smaller neurons with “stunted” dendrites are reported in ASDs [269]. Carboxyethyl pyrrole (CEP), a stable marker for oxidative stress, localizes predominantly in dendrites, across multiple brain regions in ASDs [270]. Higher intracellular concentrations of Ca^++^ in the ASD brain [271] are compatible with the excitotoxic mechanism of hypoxia. Excess intracellular Ca^++^ exerts toxicity by stimulating too much parenchymal NO [272], possibly reflected in reported nitrotyrosine elevation across multiple brain regions in ASDs [273].

## 7. Research and Treatment Implications

Abnormal baroreflex and vagal tone are the strongest objective evidence of impaired NTS function in ASDs, so the first test for the hypothesis is confirmation of these electrophysiological findings. A second strong clue, DMSA chelation studies, also requires expansion. Examination of the electrophysiological parameters in relation to response to DMSA, brain scans, and other treatments could yield a more coherent view of ASDs. Many other specific investigational and treatment approaches to ASDs are suggested by the hypothesis. 

Promising treatments such as iron [274], ascorbate [275], and hyperbaric oxygen (HBO) [276,277] fit the hypothesis mechanistically, via enhanced delivery of oxygen to the pNTS. Iron supplementation is predicted to increase oxygen-carrying capacity of blood reaching the pNTS via increased ferritin and hemoglobin levels; ascorbate supplementation could enhance blood flow at the pNTS by reducing oxidative stress and thereby reducing blood viscosity; HBO potentially increases oxygen saturation of blood that reaches pNTS. Electrophysiological changes in vitamin B6 responders [278,279] are intriguing because B6-dependent cystathionine-beta-synthase (CBS) catalyzes the production of hydrogen sulfide (H_2_S) [280], which has been shown to augment synaptic neurotransmission in the NTS [281]. H_2_S is also shown to detoxify mercury [282]. Blood viscosity testing and markers for oxidative stress could illuminate clinical presentations and therapeutic outcomes. 

Novel therapies for NTS dysfunction might include transcutaneous stimulation of the auricular vagus nerve. In animals, transcutaneous VNS blocks LPS-induced inflammation [283]. Chinese Traditional Medicine considers the Anmian (EX17) accupoint effective for vagal upregulation. Electrical stimulation of the point in animals alters sleep pattern, but the effect is prevented by precise opioid blockade of the NTS [284].

It is hoped that the field will proceed immediately to direct examination of the pNTS in tissue from subjects with ASDs, including determination of toxin levels. Parallel examination of brainstem from animals challenged with toxins and hypoxia could improve interpretation of human tissue studies. These animal studies—and future epidemiological and environmental assessments—would accommodate the hypothesis by including a full range of neurotoxic hydrophiles, including mercury, cadmium, MSG, nitrites, and fluoride as potential triggers for ASDs. 

The NTS hypothesis ought to be viewed in relation to genetics. Monoamine oxidase (MAO) polymorphism associates with ASDs [285] and might be expected to influence development or function of catecholaminergic neurons of the NTS. The gene for homeobox transcription factor, Engrailed-2 (En-2), associates with ASDs and is known to influence early development of the hindbrain and monoaminergic neurons [286]. Polymorphisms of homeoboxA1 and B1 (HOXA1, HOXB1) influence brainstem development [287]. Phosphatase and tensin homologue (PTEN) associates with ASDs and anomalous vascular development [288]. Apparently unexamined in ASDs are two other homeobox genes, Rnx and Phox2b, which are co-expressed in all newly formed NTS neurons and are essential for development of the NTS [289]. Mutant Phox2b homozygotes fail to form an NTS altogether [290]. The alpha-2A-adrenergic receptor gene (ADRA2A) is noted to associate with decreased cerebral perfusion in ADHD [291].

A viral etiology for ASDs has not been excluded [292] and evidence suggests that it must be considered going forward. In mice, the influenza A virus has been shown to enter the brain from respiratory passages via the vagus nerve [293]. Intranasal inoculation of mice resulted in pneumonia and encephalitis restricted to the brain stem and localized primarily to the NTS. Prior to the development of brain lesions, viral antigen was detected in the vagal ganglia [294]. Impermanent behavioral changes in three children with acute encephalopathic illness were considered consistent with autism, and one of the children had increases in serum herpes simplex titers [295]. A previously healthy thirty-one-year-old male with herpes encephalitis developed “...all the symptoms considered diagnostic of autism [41,42]”.

The larger vessels serving the brainstem and the NTS are uncharacterized in ASDs. The posterior inferior cerebellar artery (PICA) has a complicated embryogenesis, and is characterized by frequent anomaly. The PICA courses below the level of the foramen magnum in symptomatic Chiari malformation [296], which is possibly more common in ASDs [297], and associates with baroreflex impairment [298]. PICA anomalies include extradural origin at C1, C2 or C3 [299], and absence in 2.5% of humans [300]. The vagus nerve is subject to compression by PICA [301]. The status of these larger vessels in ASDs needs to be determined.

Advances in perinatal management have prevented huge numbers of stillbirths and have limited the extent of brain damage in countless surviving neonates. But three aspects of modern perinatal practice are problematic from the perspective of this hypothesis. The first concern is use of terbutaline for suppression of premature contractions. Terbutaline has been shown in newborn rats to impair normal development of peripheral noradrenergic neurons [302], an effect that might also be seen in the noradrenergic A_2_ neuronal subgroup of the pNTS. A second concern is the shift to more rapid clamping of the umbilical cord which began some 35 years ago over fears of excessive autoperfusion [303]. Rapid cord-clamping is just now undergoing reconsideration by obstetricians [304] and could adversely affect oxygen delivery to a sensitive structure such as the pNTS in a crucial time-frame. The third worrisome, but perhaps unavoidable, practice is the use of oxygen in the neonatal period. Neonatal rescue of rat pups with oxygen at 60% over one week substantially changed neurotrophin and caspase levels in the NTS, shifting the local molecular environment to proapoptotic [305]. Co-administration of NMDA antagonists, magnesium, or antioxidants could possibly offset these effects of neonatal oxygen. 

An unusual characteristic of the NTS that might facilitate future treatment of ASD is persistence of progenitor stem cells that confer neogenesis of both neurons and glia, even in adulthood [306]. Immunochemistry has shown that astrogliogenesis concentrates at the border of the NTS and the AP, and vagal section is shown to stimulate robust neurogenesis in the adult NTS [307]. Regenerative NTS cells in proximity to the AP appear to connect with cells lining the IV ventricle [308]. Brain-derived neurotrophic factor (BNDF) is a powerful modulator of NTS synaptic transmission, stored in and released from vagal afferent terminals [309]. Severe depletion of BNDF in the NTS was demonstrated in a mouse model of Rett syndrome [310], which is clinically similar to ASDs. Experimentally, intracerebroventricular injection of growth factors is shown to stimulate neurogenesis in the NTS of animals [308]. If pNTS pathology is significant to ASDs, then intraventricular injection of growth factors to stimulate pNTS progenitor cells is a potential treatment. 

As discussed, disordered vocalization in PD is considered secondary to abnormal laryngeal somatosensory function [173]. Diagnostic and treatment approaches in PD [311] may be of use in ASDs. Non-closure glottal patterns are demonstrable with videolaryngostroboscopy [176]. Voice-onset errors associated with vagal dysarthria may be noticeable to speech pathologists, or detectable by decreased spectrum entropy in automated speech analysis [312]. In infants, abnormal auditory brainstem response associates with laryngeal brainstem response [313], a non-invasive diagnostic modality.

## 8. Conclusions

The hypothesis inverts the prevailing neurobiological construct, which attributes ASDs to primary dysfunction of multiple regions of the higher brain, or their connections. It certainly accommodates collateral dysfunction of these higher structures, from perinatal hypoxia also affecting the NTS, or cerebrovasculature dysregulation stemming from functional impairment of the NTS. Impaired flow of viscerosensory information to the higher centers, especially via the ascending noradrenergic system, makes a significant contribution to abnormal complex behaviors of ASDs—social interaction, attention, motivation, memory, emotion, and decision-making. 

Primary impairment of NTS function by perinatal hypoxia is proposed to be a sufficient trigger for early-onset ASDs. The hypothesis posits that toxins trigger ASDs via effects on the NTS at any time in the developmental sequence, from embryonic to ambulatory. It is possible that different toxins acting independently or in combination upon this vital region of the brain are triggers of ASDs. Toxic effects on the NTS, including impaired oxygenation, are potentially reversible. We emphasize that specific toxins preferentially enter the pNTS, and are common in food, drink, and the very air we breathe. 

The neuropsychological substrate for the hypothesis is provided by Antonio Damasio [314] and Emeran Mayer [315], who consider visceral afference as the necessary basis of complex behaviors and sense of self. The term “autism”, from the Greek, “autos”, for self, describes children thought to be cut off from the outside world. Time will tell to what extent they are separated from their inner, visceral world by pathology at the pNTS. 

## Glossary of Abbreviations

5-HT: Serotonin
ACh: Acetylcholine
AChE: Acetylcholinesterase
ADHD: Attention Deficit Hyperactivity Disorder
AP: Area Postrema
ASD: Autism Spectrum Disorder
BBB: Blood-Brain Barrier
CBF: Cerebral Blood Flow
CO: Carbon Monoxide
CSB: Cardiac Sensitivity to Baroreflex
CSF: Cerebrospinal Fluid
CVO: Circumventricular Organ
CVT: Cardiac Vagal Tone
DBP: Diastolic Blood Pressure
DMSA: Dimercaptosuccinic Acid
DMV: Dorsal Motor Nucleus of the Vagus X
GCF: Glottal Closing Force
HBO: Hyperbaric Oxygen
HR: Heart Rate
LPS: Lipopolysaccharide/Endotoxin
MAP: Mean Arterial Pressure
MSG: Monosodium Glutamate
MT: Metallothionein
NE: Norepinephrine
NO: Nitric Oxide
NTS: Nucleus Tractus Solitarius
PD: Parkinson’s Disease
pNTS: permissive region of NTS
RSA: Respiratory Sinus Arrhythmia
SHR: Spontaneously Hypertensive Rat
TH: Tyrosine Hydroxylase
TNF: Tumor-necrosis Factor alpha
VNS: Vagal Nerve Stimulation
